# Digital assay for rapid electronic quantification of clinical pathogens using DNA nanoballs

**DOI:** 10.1126/sciadv.adi4997

**Published:** 2023-09-06

**Authors:** Muhammad Tayyab, Donal Barrett, Gijs van Riel, Shujing Liu, Björn Reinius, Curt Scharfe, Peter Griffin, Lars M. Steinmetz, Mehdi Javanmard, Vicent Pelechano

**Affiliations:** ^1^ Rutgers, The State University of New Jersey, New Brunswick, NJ, USA.; ^2^ SciLifeLab, Department of Microbiology, Tumor and Cell Biology, Karolinska Institutet, Solna, Sweden.; ^3^ International Institute of Tea Industry Innovation for the Belt and Road, Nanjing Agricultural University, Nanjing 210095, China.; ^4^ Department of Medical Biochemistry and Biophysics, Karolinska Institutet, Solna, Sweden.; ^5^ Yale University, New Haven, CT, USA.; ^6^ Stanford Genome Technology Center, Stanford, CA, USA.; ^7^ Department of Genetics, Stanford University School of Medicine, Stanford, CA, USA.

## Abstract

Fast and accurate detection of nucleic acids is key for pathogen identification. Methods for DNA detection generally rely on fluorescent or colorimetric readout. The development of label-free assays decreases costs and test complexity. We present a novel method combining a one-pot isothermal generation of DNA nanoballs with their detection by electrical impedance. We modified loop-mediated isothermal amplification by using compaction oligonucleotides that self-assemble the amplified target into nanoballs. Next, we use capillary-driven flow to passively pass these nanoballs through a microfluidic impedance cytometer, thus enabling a fully compact system with no moving parts. The movement of individual nanoballs is detected by a change in impedance providing a quantized readout. This approach is flexible for the detection of DNA/RNA of numerous targets (severe acute respiratory syndrome coronavirus 2, HIV, β-lactamase gene, etc.), and we anticipate that its integration into a standalone device would provide an inexpensive (<$5), sensitive (10 target copies), and rapid test (<1 hour).

## Teaser

Electronic detection of self-assembled DNA nanoballs enables simple label-free pathogen detection.

## INTRODUCTION

Fast and accurate detection of genetic material is a cornerstone for diagnostics with far-reaching applications, including the identification of infectious diseases and patient biomarkers (*1*–*3*). The recent severe acute respiratory syndrome coronavirus 2 (SARS-CoV-2) pandemic has led to a resurgence of diagnostics methods targeting viral nucleic acids or proteins (*4*). Because of their intrinsic simplicity and scalability, the use of protein-based diagnostics such as rapid antigen detection coupled with lateral flow has been widely applied (*5*). However, protein-based tests require the development of high-quality antibodies and were thus deployed relatively late into the pandemic (*6*). Nucleic acid–based approaches are in general easier to develop, with higher sensitivity and intrinsic flexibility (*7*).Reverse transcription quantitative polymerase chain reaction (RT-qPCR) is the gold standard for nucleic acid detection and has been widely applied to diagnose SARS-CoV-2 (*7*, *8*). To complement RT-qPCR, several other nucleic acid–based approaches have been developed (*7*). Of particular relevance are the isothermal amplification approaches that offer simple implementation and scalability even in resource limited settings. Among those, loop-mediated isothermal amplification (LAMP) is one of the most popular approaches (*9*). The LAMP reaction uses four to six oligonucleotides targeting a region of interest together with a strand-displacing polymerase. As it does not require a thermocycler, it is a simple, low-cost, yet sensitive alternative to the standard PCR (*10*). LAMP combined with a reverse transcriptase (RT-LAMP) has been widely used for pathogen detection (*11*, *12*) including SARS-CoV-2 (*13*). DNA detection after LAMP can be achieved by multiple strategies including gel electrophoresis and fluorescent and colorimetric methods (*7*, *9*, *14*). Fluorescent methods while successfully deployed increase reagent cost and require complex readout systems or integration with dedicated platforms (*15*, *16*). As an alternative, colorimetric methods provide a simple visual color change in response to H^+^ ion production. However, this approach is susceptible to false positives; e.g., raw saliva can be acidic and can require additional steps before testing to neutralize such samples (*17*, *18*). Thus, the development of simplified methods for detection of nuclei cell acid amplification is desirable.

Electrochemical detection of DNA amplification via measuring impedance changes is a label-free alternative to fluorescent and colorimetric techniques. Here, the material of interest is subjected to an excitation voltage at specific frequencies, and the response that is dependent on the impedance of the material is recorded (*19*). Impedance-based biosensors have been used for a wide range of analytes including proteins (*20*), nucleic acids (*21*), and cancerous cells (*22*) with a high degree of sensitivity (*19*). Moreover, electronic components have undergone rapid miniaturization due to technological advancements in recent decades (*23*), allowing for increased portability and relatively inexpensive production. In addition, substantially reduced volume input requirements further advance the possibility for point-of-care (POC) detection (*24*). We have previously successfully quantified DNA (*25*) of varying concentrations and lengths using impedance-based detection in a microfluidic chip. However, that approach required the use of microbeads. This increases cost and complexity, which would preclude its use in low-resource settings. Impedance-based detection of DNA molecules in solution has been demonstrated (*26*); however, such detection could not distinguish between different DNA or RNA target sequences. Thus, there is a need for novel one-pot approaches using label-free impedance-based diagnostics making use of the many advantages of electrical-based detection (e.g., fast, accurate, small, cheap, scalable, etc.) for specific target sequences bringing us closer to a POC setting.

Here, we developed a simplified one-pot isothermal amplification of nucleic acids that can be detected by a change in electrical impedance without the need of microbeads as external nucleation agents. We modified RT-LAMP reactions to self-nucleate the amplified DNA into nanoballs that can be detected by an inexpensive and simple electrical detection system. We show that the passive flow of the generated self-assembling DNA nanoballs past two detection electrodes in a simple microfluidic channel generates quantized spikes in the impedance signal. To demonstrate the applicability of this technology, we apply it to the detection of SARS-CoV-2. The number of detected spikes corresponds to the amount of amplified DNA and thus higher viral concentrations. We subsequently demonstrate detection of multiple DNA and RNA pathogenic sequences including HIV, influenza, *Mycobacterium tuberculosis*, and β-lactamase from various viral and bacterial sources, which paves the way for a novel and simple impedance-based multipathogen POC nucleic acid detection system.

## RESULTS

### Electric detection of self-assembled DNA nanoballs

**Fig. 1.**
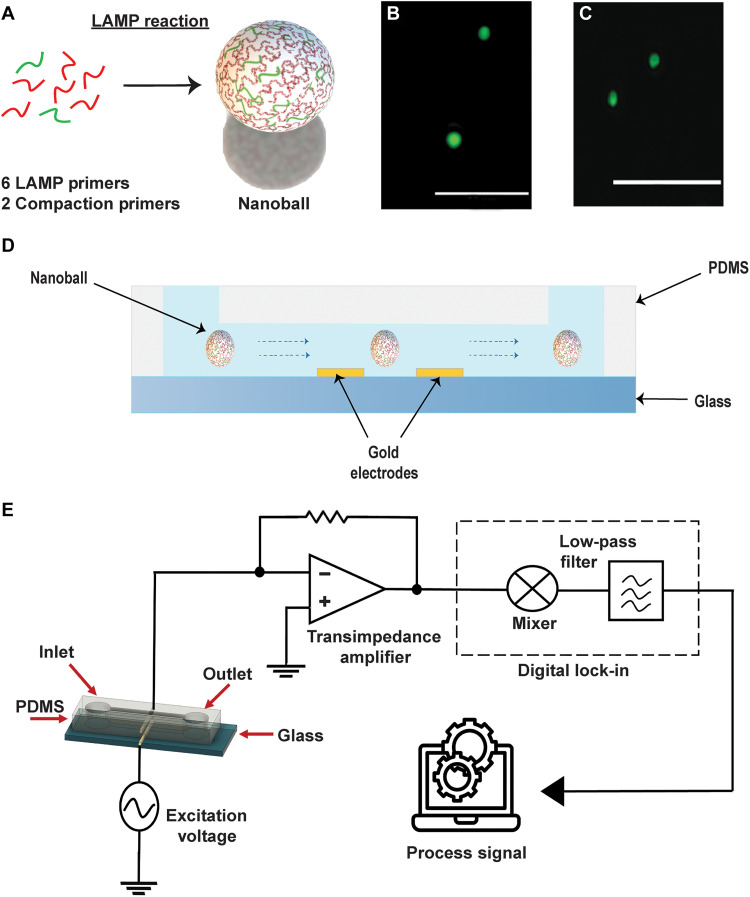
Electrical detection of DNA nanoballs. (**A**) Formation of DNA nanoball using six target-specific LAMP oligos and two compaction oligos. (**B**) Fluorescence image of DNA nanoballs. Scale bar, 10 μm. (**C**) Fluorescent image of 1 μM MyOne Dynabeads as size reference. Scale bar, 10 μm. (**D**) Passive flow of DNA nanoballs in a microfluidic chip made of PDMS on a glass substrate integrated with gold electrodes. The passage of DNA nanoballs through the gold electrodes occludes the current path and disturbs the electric field formed between the gold electrodes. (**E**) A schematic illustrating the electronic readout system used for the microfluidic chip with integrated gold electrodes.

To simplify impedance-based detection of DNA (*25*), we first had to develop a molecular approach able to generate DNA nanoballs in a one-pot reaction and without the need of external nucleation agents. Inspired by previous work on rolling circle amplification (RCA) (*27*), we modified LAMP reactions to self-nucleate the amplified DNA into nanoballs. During a standard LAMP amplification, a series of concatemer products of different lengths are generated. Although LAMP amplicons are much shorter and more heterogeneous in size than those generated by RCA, we reasoned that we could use oligonucleotides complementary to a common region present in the amplicons to “staple” them together into a DNA nanoball (compaction oligos, see table S1). In addition, those oligos could be already present in the LAMP reaction enabling the generation of the DNA nanoballs in parallel to the isothermal DNA amplification (Fig. 1A). In total for a given target sequence, our reaction contains the standard six target-specific LAMP primers and two additional compaction oligos. To test this novel concept, we decided to apply it to the detection of SARS-CoV-2 RNA. We modified the standard As1e (*28*) RT-LAMP Loop F (LF) and Loop B (LB) primers targeting the SARS-CoV-2, introducing two repeats to generate a first version of our compaction oligos (see table S1). Next, we tested this modified RT-LAMP reaction using synthetic SARS-CoV-2 RNA as we have previously done (*13*) (see Materials and Methods for details). We were able to generate self-assembling compact DNA nanoballs ranging from 1 to 2 μm in diameter, which could be observed under a fluorescent microscope (Fig. 1, B and C). We then proceeded to test whether we could detect these DNA nanoballs by impedance detection in our microfluidic device, as illustrated in Fig. 1 (D and E).

**Fig. 2.**
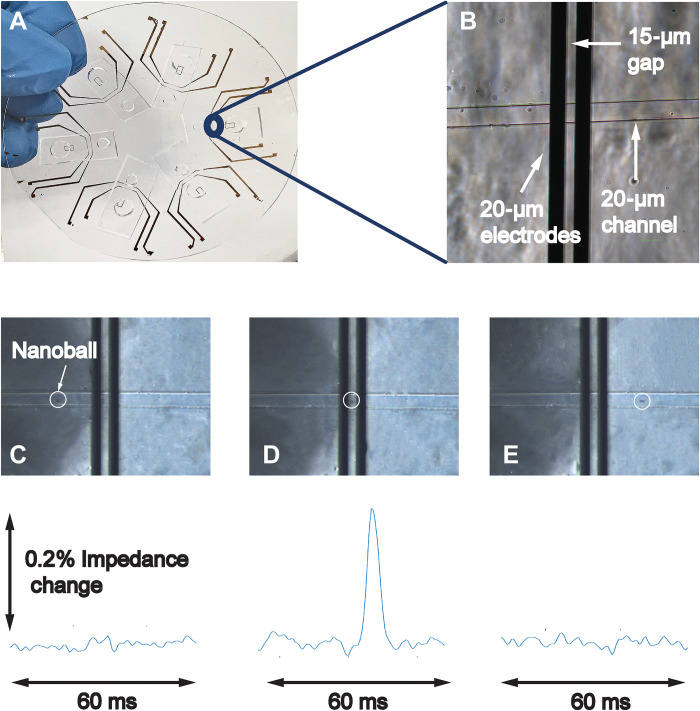
Microfluidic chip for impedance-based detection of DNA nanoballs. (**A**) A photograph of the 3-inch fused silica wafer with six microfluidic devices. (**B**) Microscopic image of the channel with the integrated gold electrodes. (**C** to **E**) Principle of detection of DNA nanoballs. The passing of a DNA nanoball through the integrated gold electrodes produces a spike signature in the impedance response of the system. This impedance response is recorded as a single DNA nanoball.

We used a simple microfluidic chip made of polydimethylsiloxane on a glass substrate with gold electrodes (a photograph and microscopic image can be seen in Fig. 2, A and B, respectively). The microfluidic chip uses passive flow driven by capillary action, thereby eliminating the need of complex microfluidic tubes and pumps. This simple passive flow moves the DNA nanoballs through the channel above gold electrodes (Fig. 1D) connected to an electronic readout system (Fig. 1E). As a nanoball passes the gold electrodes, it occludes the current path and the electric field between the two electrodes. This generates a change in impedance, resulting in a peak signature at the output of the readout system similar to the one shown in Fig. 2 (C to E).

**Fig. 3.**
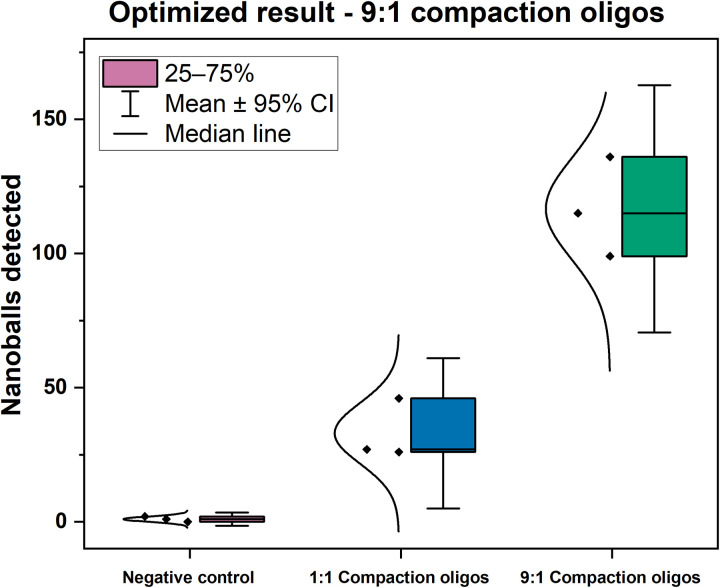
Optimization of the required ratio of compaction oligos. Box plot of number of nanoballs detected in 1:1 compaction oligos versus 9:1 compaction oligos with deionized water as negative control using two-repeat compaction oligos and 100,000 copies of synthetic SARS-COV-2 RNA as template. The error bars represent a 95% confidence interval (CI). Three separate LAMP reactions were performed followed by independent microfluidic measurements.

The initial RT-LAMP compaction experiments revealed the formation of numerous, distinct, and regular ball-like structures comparable in size to 1-μm beads as shown in Fig. 1C. Next, we experimented with varying the concentration of compaction oligos in relative to LF and LB primers (either 1:1 ratio or 9:1 ratio of compaction versus amplification oligos; see Materials and Methods for details). The use of a higher proportion of compaction oligos (9:1) yielded a higher average number of detected DNA nanoballs (>100 in 10 min) (Fig. 3B), and therefore, we used this ratio for the following experiments.

### Optimization of impedance detection of DNA nanoballs

Once we demonstrated our ability to generate DNA nanoballs in a one-pot reaction, we optimized the electrical detection of the nanoballs in the microfluidic system. We tested different configurations by varying the parameters of the electrical detection system such as excitation voltage, excitation frequency, transimpedance gain, and the low-pass filter bandwidth. The results of these parameter permutations are summarized in table S2 where we used standardized 1-μm Dynabeads in our microfluidic chip, identifying an optimal configuration. The Dynabeads act as a proxy for the DNA nanoballs and produce a similar impedance signal, while stock solutions of Dynabeads allow dilution experiments to be performed in a controlled manner. We measured the following parameters to optimize the sensitivity of the system: number of spikes detected, the baseline voltage, the signal-to-noise ratio, and the spike voltage. The results of these optimizations are summarized in fig. S1. We found out that a high excitation voltage correlates to a higher spike voltage and higher number of spikes detected for the same concentration of Dynabeads. However, too high of a voltage can damage the electrodes and lead to hydrolysis and consequently electrical breakdown. Therefore, we identified 5 V to be the optimal excitation voltage. We also found out that increasing the frequency yielded improvement up to 5 MHz. Hence, we use 5 MHz as the excitation frequency. Last, we confirmed that our performed optimization of impedance-based detection using Dynabeads lead also to an improved detection of DNA nanoballs. Using these refined settings, we saw a substantial improvement in the number of DNA nanoballs detected. For equivalent synthetic RNA input (10^5^), detection went from less than 50 DNA nanoballs (in form of spikes in the electric signal) to an average of approximately 2000 (see fig. S2).

### Limit of detection using a dilutions series

**Fig. 4.**
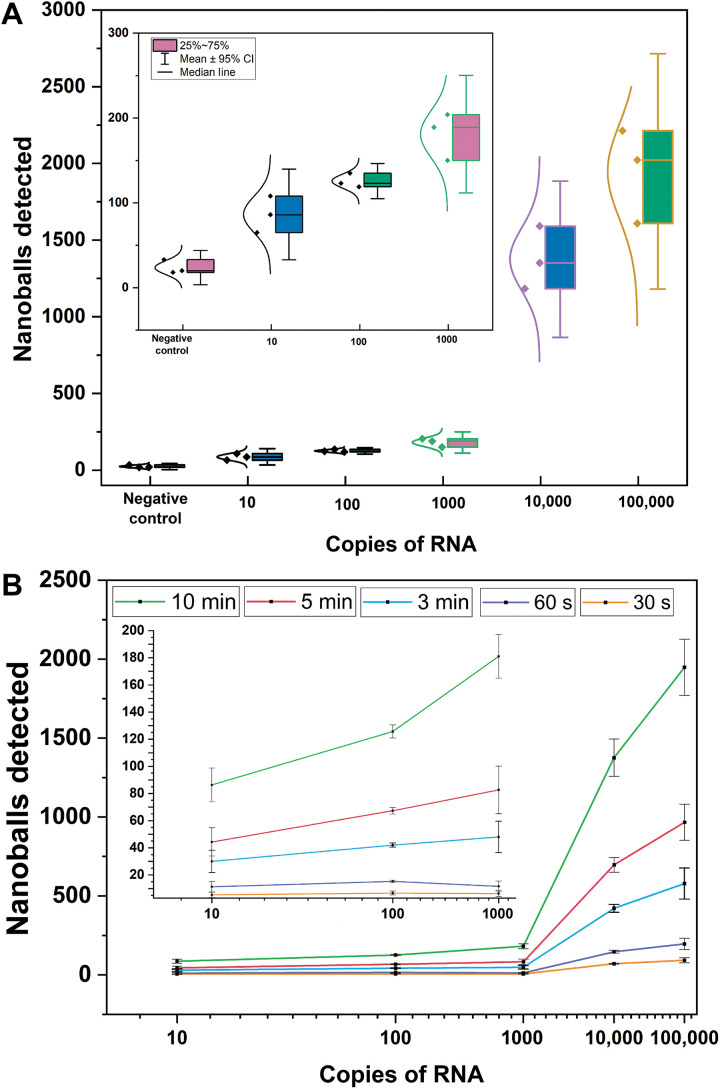
Analysis of the limit of detection. (**A**) Boxplot for the dilution series experiment to determine the limit of detection using two compaction oligos and 9:1 ratio. The error bars represent 95% CI. **(B)** Nanoballs detected for different assay times. The error bars represent a 95% CI. All conditions done in triplicate.

After these improvements we explored the limit of detection (LOD) of our method. We carried out a dilution series experiment using synthetic RNA as template in the RT-LAMP compaction reaction. Next, we used our impedance microfluidic system to detect nanoballs for a period of 10 min (Fig. 4A). We observed from these data that even the lowest concentration of synthetic RNA tested (10 copies of RNA) was easily distinguishable from negative controls. We also tested the effect of shorter electric detection time on our LOD. We analyzed the data from the 10-min experiments using our optimized configuration (compaction oligos with two repeats and a 9:1 ratio) at various time intervals (5 min, 3 min, 60 s, and 30 s). Even at 30 s, the distribution of number of nanoballs detected correlated well to the concentration of the initial synthetic RNA spiked into the reaction and exhibiting statistically significant difference between the negative control group (Fig. 4B).

### Three-repeat compaction oligo design leads to enhanced detection

**Fig. 5.**
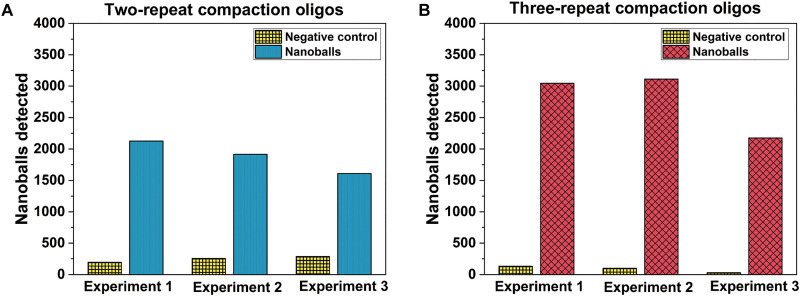
Optimization of experimental protocol for the detection of DNA nanoballs. (**A**) Two-repeat compaction oligos. (**B**) Three-repeat compaction oligos with 100,000 copies of synthetic SARS-COV-2 RNA as template.

To further improve the DNA detection ability of our system, we used several strategies. We reasoned that attempting to increase the size of the DNA nanoballs or changing their electrical properties may increase the signal-to-noise ratio and thus improve detectability. To increase DNA nanoball size, we reasoned that adding an additional copy of the common sequence to the compaction oligos may enhance the ability of the LAMP amplicons to compact and form nanoballs (three-repeat compaction oligos; see table S1). The use of the three-repeat compaction oligo repeats in a standard 9:1 ratio substantially increased the number of average DNA nanoballs detected from approximately 2000 when using two-repeat compaction oligos to close to 3000 (Fig. 5).

### Impedance-based detection of SARS-CoV-2 in clinical samples

**Fig. 6.**
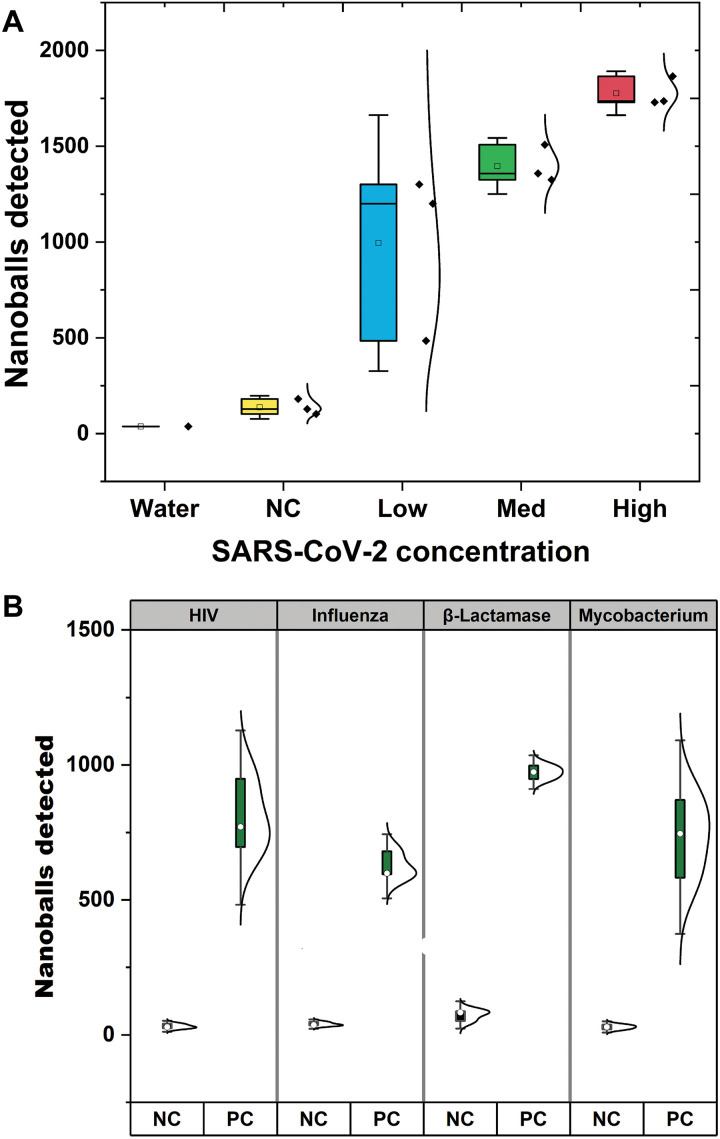
Testing clinical samples for patients with COVID-19 and multiple pathogenic sequences. (**A**) Nanoballs detected from heat-inactivated nonextracted residual specimens of SARS-CoV-2–positive and SARS-CoV-2–negative nasopharyngeal samples previously confirmed by RT-PCR. The samples were divided into four groups: 5 SARS-COV-2–negative samples (NC2, yellow), 15 SARS-CoV-2–positive samples divided into three subgroups on the basis of RT-qPCR–estimated viral load: low (blue, Ct 26–27), medium (green, Ct 22–24), and high viral load (red, Ct 17–20). Negative controls (samples tested negative by RT-qPCR) are in yellow and a water control in purple. The error bars represent the SD for each group. (**B**) Boxplot showing the number of nanoballs detected for negative controls (NC) and positive controls (PC) for HIV, influenza, Mycobacterium, and β-lactamase. The error bars represent 95% CI.

To test the applicability of our approach, we next applied it to detect SARS-COV-2 from upper-airway swab samples sourced from clinical diagnostic testing. We used surplus aliquots of heat-inactivated upper-airway samples (combined throat swab, nose swab, and saliva) clinically diagnosed with COVID-19 by RT-PCR (*29*) as well as negative samples (see Materials and Methods and table S3). We randomly selected five samples across different viral loads as measured by RT-qPCR: low (Ct 26–27), medium (Ct 22–24), and high viral load (Ct 17–20). In addition, we included five samples that were tested negative for COVID-19 by clinical RT-qPCR. We ran our modified RT-LAMP directly on these samples together with our results (Fig. 6A), importantly demonstrating the ability of our impedance-based detection to detect clinical relevant viral levels in nonextracted heat-inactivated samples. In agreement with our earlier optimization trials in which we spiked in synthetic SARS-COV-2 RNA, we observed a strong relationship between viral load in the samples and number of detected DNA nanoballs. Here, we detected between 1000 and 2000 DNA nanoballs across the range of Ct values (27 to 17). Of further importance is the low levels of impedance peaks detected from SARS-CoV-2–negative patients despite the complex material (combined throat swab, nose swab, and saliva) in these samples (Fig. 6A).

### Flexible detection of pathogens using DNA nanoballs

To demonstrate the flexibility and ease of use of our LAMP compaction approach combined with impedance detection, we designed LAMP compaction oligos against multiple pathogens using as a base previously published LAMP designs (*30*–*33*). We designed three-repeat compaction oligos to target regions used to diagnose pathogens that pose a threat to public health especially in low-income regions. These included oligos against *M. tuberculosis* (*30*), HIV (*31*), Influenza-A/H1N1 (*32*), and a common β-lactamase producing antimicrobial resistance gene (*33*) (table S4). As shown in Fig. 6B, we successfully detected DNA nanoballs when performing RT-LAMP (or LAMP) compaction reactions using synthetic genetic material of the selected pathogens (table S5). All targets showed close to 1000 peaks detected and significantly different from negative water controls. This demonstrates the ease of adapting any LAMP reaction to a “LAMP compaction” strategy coupled with impedance-based detection.

## DISCUSSION

Here, we describe a novel modified LAMP amplification reaction with two additional compaction oligos that produces self-assembling DNA nanoballs in a one-pot reaction. Using gravity-driven flow of the nanoballs passing electrodes in a microfluidic channel, the number of nanoballs produced can be accurately counted using impedance-based electrical detection providing label-free, quantized DNA measurements of the viral load. We performed a proof of concept focusing on the application of this technology to the detection of SARS-CoV-2. Our modified isothermal RT-LAMP reaction with compaction oligos also worked directly on heat-inactivated, nonextracted samples as we and others previously demonstrated with standard RT-LAMP (*13*). Furthermore, we extended our system to detect various pathogens of both viral and bacterial origin, from as little as 10 original target copies proving proof of principle of targeting a wide variety of pathogens in a sensitive manner. In contrast to protein-based detection, such as rapid antigen tests that rely on antibody production (*5*), a nucleic acid–based approach allows for greater flexibility and easy modification to detect various targets. Further, while here we used a 60-min RT-LAMP reaction followed by impedance-based detection, RT-LAMP reaction times can be easily decreased (*13*) to enable faster diagnostics.

The COVID-19 pandemic has highlighted the need for simplified methods for nucleic acid–based detection. Most currently used methods rely on fluorescence or colorimetric indicators (*9*, *14*) that may increase cost, complexity, and, in some cases, lead to false positives and limited scalability (*15*, *18*). A general limitation of isothermal amplification is their propensity to provide a higher-level false-positive (background) amplification in respect to RT-qPCR (*34*). This intrinsic propensity can be controlled by carefully designing the used primers (*28*). In our implementation of RT-LAMP compaction, only produced amplicons containing complementary sequences to the compaction oligos would self-assemble into DNA nanoballs and thus would be detected. To maximize specificity, we designed our novel RT-LAMP compaction strategy based on well-established RT-LAMP oligos against SARS-CoV-2 (*28*). While our results are promising, a true estimation of specificity would require a much larger number of healthy individuals. However, by combining the specificity provide by RT-LAMP with the one provided by DNA nanoball assembly, it can be expected that this novel strategy will provide higher specificity than standard RT-LAMP.

Electrical detection of DNA has been previously explored (*25*); while both robust and effective, the need for conjugation of DNA to beads increases cost and precludes scalability. The electrical characterization of DNA in solution has also been performed using an impedance sensor (*26*). These measurements were, however, not related to a specific DNA sequence in contrast to our targeted approach with a quantized readout. A limitation of our current implementation is the need for a benchtop impedance measurement spectroscope. However, in this work, we have demonstrated that DNA nanoball detection can be performed using a single voltage, frequency, and amplifier setting. An additional advantage of the presented approach is that, by removing the need to use microbeads as nucleating agents for impedance-based detection of DNA (*25*), our label-free approach would be, in principle, amenable to reagent lyophilization. In addition, our assay can provide a quantized readout and is not susceptible to false positives due to the pH of raw samples, e.g., saliva (*17*, *18*). Thus, by combining the use of affordable mass-produced electronics with lyophilized reagents, the technology that we present here has the potential to serve as a basis for the development of a low-cost (<$5), widely deployed, and scalable POC device (*35*). Because our current implementation operates on capillary flow and does not require pumps or actuators, we envision a future device consisting of two modules: a reaction chamber providing an isothermal temperature of 65°C and an affordable impedance detector operating by capillary flow. It would be advantageous and feasible to also combine both the heated RT-LAMP incubation (e.g., via a small battery) and the subsequent impedance-based detection in one standalone device.

In summary, we demonstrated a passive flow impedance–based detection of novel label-free DNA nanoballs targeting SARS-CoV-2 and later various pathogens of both viral and bacterial origin. It potentially provides a sensitive (10 copies), cheap, fast (<60 min), and scalable POC system to help address the growing pathogen detection challenges in the coming decades.

## MATERIALS AND METHODS

### RT-LAMP primers, compaction oligonucleotide design, and synthetic RNA/DNA–positive controls

We modified previously As1e primers designed against the orf1ab region of SARS-CoV-2 viral genome to make “compaction oligos.” For two of the six standard As1e primers, namely, As1 LF and As1 LB (see table S1), we duplicated the oligo sequence placing a 3-nt AAA linker sequence between each to form a “compaction” oligo for each. For the first compaction oligo designs and optimization experiments, we included two such repeat sequences with one spacer (so-called two-repeat compaction oligos) as well as modifying with or without a three-prime inverted dT nucleotide modification. In later optimizations described below, we added a further repeat (three-repeat compaction oligos) of the original sequence and a second AAA spacer (see table S1). To track potential nanoball formation with fluorescence microscopy, we added a 5′ fluorophore modification: 5′ 6 FAM (fluorescein) to the standard As1 F3 primer. We also modified established RT-LAMP primers to produce three-repeat compaction oligos against *M. tuberculosis* (*30*), HIV (*31*), Influenza-A/H1N1 (*32*), and a common β-lactamase–based antimicrobial resistance gene (*33*) (see table S4). All oligonucleotides were purchased from IDT (Integrated DNA Technologies, 1710 Commercial Park Coralville, IA 52241 USA) with standard desalting and dissolved in nuclease-free water upon arrival. We investigated various versions of our compaction oligos including 3′ modifications (invdT) to prevent oligo extension and polymerization in attempts to favor compaction. Our first compaction oligo designs included so-called “two-repeat” compaction oligos.

We used synthetic fragments of SARS-CoV-2-RNA generated as previously described (*13*) by in vitro transcription of PCR fragments with sequences including a T7 promoter and a part of SARS-CoV-2 sequence targeted by As1e primers. We amplified the PCR product using T7-HMS1-FW(TAATACGACTCACTATAGGGTGCTTGTGAAATTGTCGGTGGA) and HMS1_rv (GCTTTTAGAGGCATGAGTAGGC). The synthetic RNA was produced using the TranscriptAid T7 High Yield Transcription Kit (Thermo Fisher Scientific, Waltham, MA, USA), then deoxyribonuclease-treated with a TURBO DNA-free kit (Thermo Fisher Scientific, Waltham, MA, USA), and subsequently purified with Ampure XP beads (Beckman Coulter, Brea, CA, USA). RNA was quantified using a Qubit RNA HS Assay kit (Thermo Fisher Scientific, Waltham, MA, USA).

To generate synthetic targets for the four non–SARS-CoV-2 gene targets mentioned above, we ordered pairs of single-stranded DNA oligonucleotide ultramers from IDT (1710 Commercial Park Coralville, IA 52241, USA) coding for regions targeted by their respective RT-LAMP primers. Each ultramer in a pair coded for one of the two strands of a target region with a short overlapping region in each 3' end, which facilitated subsequent annealing and Taq polymerase extension to produce a double-strand DNA fragment. We amplified these double-stranded DNA ultramers further with target-specific PCR primers (see table S5 for details) that contained either a T7 or T3 promoter sequence in the 5' region. We produced single-stranded RNA from these targets as previously described via the TranscriptAid T7 High Yield Transcription Kit (Thermo Fisher Scientific, Waltham, MA, USA). Ultramers were produced via standard desalting and dissolved in nuclease-free water.

### LAMP combined with a reverse transcriptase

See Supplementary Methods for details.

### SARS-CoV-2 clinical samples

See Supplementary Methods for details.

### Fluorescent microscopy imaging

See Supplementary Methods for details.

### Microfluidic chip

See Supplementary Methods for details.

### Experimental setup for impedance-based detection

The gold electrodes from the microfluidic chip are connected to a commercial benchtop Impedance Spectroscope (Zurich Instruments, HF2IS). The microfluidic chip is placed inside a Faraday cage to minimize noise and interference. An excitation voltage is applied across the electrodes at a programmable frequency by connecting one electrode to the impedance spectroscope directly whereas the other electrode is connected to a transimpedance amplifier (Zurich Instruments, HF2TA) with a programmable transimpedance gain. The output of the transimpedance amplifier is fed back into the impedance spectroscope for demodulating and filtering the signal. The parameters of the impedance spectroscope were optimized, and we used an excitation voltage of 5 V at a frequency of 5 MHz with a transimpedance gain of 1 kilohm and a bandwidth (low-pass filter cutoff frequency) of 100 Hz. The signals are stored on a PC and are processed using MATLAB.

### DNA nanoball detection algorithm

The data obtained from the measurements are processed using an algorithm implemented using MATLAB. The data obtained from the impedance spectroscope typically has a baseline voltage and drift associated. Firstly, the baseline of the signal is computed using a moving average filter. This baseline signal is then subtracted from the original signal to have a normalized signal with only the peaks/spikes present. These peaks represent a change in impedance for a very short duration due to a nanoball or a bead passing through the microfluidic chip across the electrodes. This signal is passed through a filter to remove the background noise, and a threshold is applied for the detection of peaks in the response. This threshold is kept 2 μV above the noise. The noise of the signal is computed using the variation of the response in the control group when there are no peaks in the response. The outliers are then removed from the peaks and the number of peaks detected are noted as the number of nanoballs detected by the system.

### Optimization of electrical parameters of the impedance spectroscope

See Supplementary Methods for details.
