# Supplementary material for: Digital assay for rapid electronic quantification of clinical pathogens using DNA nanoballs
Source: Sci Adv. 2023 Sep 6;9(36):eadi4997. doi: 10.1126/sciadv.adi4997 (PMC10482329; doi:10.1126/sciadv.adi4997)
Supplement: Supplementary file 1 — Supplementary Methods Table S1 to S5 Figs. S1 to S3 [file sciadv.adi4997_sm.pdf]

Science Advances
AAAS

Supplementary Materials for

Digital assay for rapid electronic quantification of clinical pathogens using DNA nanoballs

Muhammad Tayyab *et al.*

Corresponding author: Lars M. Steinmetz, lars.steinmetz@stanford.edu;
Mehdi Javanmard, mehdi.javanmard@rutgers.edu; Vicent Pelechano, vicente.pelechano.garcia@ki.se

Sci. Adv. **9**, eadi4997 (2023)
DOI: 10.1126/sciadv.adi4997

This PDF file includes:

Supplementary Methods
Tables S1 to S5
Figs. S1 to S3

Supplementary materials

Supplementary methods

RT-LAMP

RT-LAMP reactions were assembled in 20ul reactions on ice in PCR multistrips (Sarstedt, Numbrecht, Germany). Reactions consisted of 10ul of WarmStart Colorimetric LAMP 2X Master mix with UDG (New England Biolabs, Ipswich, MA, USA), 1ul of sample (synthetic RNA/DNA or nuclease free water for negative controls) 7ul nuclease free water and 2ul 10x LAMP primer mix. The standard primer mix consisted of 2uM F3-5'-6-FAM, 2uM B3, 16uM FIP, 16uM BIP, 4uM LF and 4uM LB. For compaction oligo optimization trials, we used a 1:1 ratio between standard LF or LB to compaction oligos (2uM LF Compaction: 2uM LF and 2uM LB Compaction oligo : 2uM LB) or a 9:1 ratio (3.6uM LF compaction oligo : 0.4uM LF and 3.6uM LB compaction oligo : 0.4uM LB). All conditions were carried out in triplicates. Reactions were at 25°C 10 min, 65°C 60 min (See Fig S3 for a simplified overview).

SARS-CoV-2 clinical samples

We obtained anonymized or pseudo anonymized surplus aliquots from 30 SARS-CoV-2 positive and 10 negative nasopharyngeal samples that had previously been clinically diagnosed for COVID-19 by RT-PCR in early February 2022 by demand of the Public Health Agency of Sweden. Specimens, originating from central Sweden, were collected in a fixed volume of 1 mL physiological saline (0.9% NaCl) and inactivated by heat (70°C for 50 min) upon arrival to the laboratory, and subsequently subjected to extraction-free SARS-CoV-2 RT-PCR. Samples were stored at 4 °C prior to use. The SARS-CoV-2 RT-PCR assay was an improved multiplex version of the extraction-free protocol developed by Smyrlaki *et. al.* (8) with increased sample input and reaction volume and increased sensitivity (29). For each reaction, 24 uL RT-PCR master mix was prepared, containing 7.5 µL TaqPath 1-Step RT-qPCR Master Mix, CG (Thermo Fisher, containing ROX as passive reference), 0.9 µL 10% Tween20 (Sigma), N1 primer-probe mix (forward: GACCCCAAAATCAGCGAAAT, reverse: TCTGGTTACTGCCAGTTGAATCTG, probe: FAM-ACCCCGCATTACGTTTGGTGGACC-BHQ1, Integrated DNA Technologies), S primer-probe mix (forward: ATATTCTAAGCACACGCCTATTATAG, reverse: CTACCAATGGTTCTAAAGCCGAA, probe: Cy5-GAGCCAGAAGATCTCCCTCAGGGT-BXQ2, Merck), RNaseP primer-probe mix (forward: AGATTTGGACCTGCGAGCG, reverse: GAGCGGCTGTCTCCACAAGT, probe:HEX-TTCTGACCTGAAGGCTCTGCGCG-BHQ1, Merck; detecting the Omicron BA.1 sub lineage), and nuclease free water up to 24 uL. Primer/Probe concentrations in the final reactions were 246/62 nM (N1), 491/125 nM (S), and 122/37 nM (RNaseP). For RT-PCR testing, 6 µL heat-inactivated nasopharyngeal swab sample (in 0.9% NaCl) was added to optical 96-well PCR plates (EnduraPlate, Applied Biosystems) containing 24 µL master mix. RT-PCR was performed on QuantStudio real-time PCR machines (Applied Biosystems) using

the QuantStudio Design & Analysis Software v1.5.2 and temperature cycles: 25 °C for 2 min, 50 °C for 10 min, 95 °C for 2 min, and 40 cycles of 95 °C for 3 s and 56 °C for 30 s. RT-PCR CT values are available in Table SYZ. Informed consent for the use of anonymized/ pseudo anonymized surplus aliquots obtained in routine clinical diagnostics was not obtained and not required, which is in accordance with the study permit obtained by the Swedish Ethical Review Authority (Dnr 2020-01945 and 2022-01139-02, Etikprövningsmyndigheten). All SARS-CoV-2 positive samples (n=30) used in our study had their viral genome sequenced using the Illumina COVIDSeq Test kit (Illumina) (See Table **S3.**for variant classifications, and Lentini *et. al.* (29) for details regarding sequencing and data analysis).

Fluorescent Microscopy imaging

To verify the production of DNA Nanoballs from our modified RT-LAMP reaction we subsequently imaged the products on a Nikon ECLIPSE Ti inverted research microscope using Plan Apo I 100x oil Ph3 DM (1.45 NA). 1-2ul per sample was pipetted onto a glass slide, allowed to air dry and a cover slip applied. 5-6-FAM (Fluorescein) fluorescence based images were captured using excitation and emission filters for GFP. Additionally, 1ul of the 1uM Dynabeads™ MyOne™ Streptavidin T1 (Thermo Fisher Scientific, Waltham, MA, USA) was imaged as a size reference.

Microfluidic chip

The microfluidic chip is made of PDMS on a glass surface with integrated gold electrodes. The first step for the formation of the microfluidic chip is patterning and fabricating the electrodes on the glass wafer. Electrodes are fabricated on glass using standard photolithography on a 3″ fused silica wafer. The process consists of photo-patterning resist on the fused silica wafer, electron beam metal evaporation, and liftoff processing. The process of photo-patterning includes wafer cleaning, spin coating the photoresist, soft bake of the resist, ultraviolet light exposure through a chromium mask printed on a 4″ × 4″ glass plate, resist development, and hard bake of the resist. Following the photo patterning process, a 100-nm-gold layer is deposited on the substrate using electron beam evaporation. A 10-nm layer of chromium is used to enhance the adhesion of gold to the glass wafer; otherwise, the gold film gets peeled off easily. We chose gold as the electrode due to its resistance to corrosion and its inert nature. The width of the electrodes was 20 µm and spacing between the two electrodes was 20 µm.

We fabricated the microfluidic channel itself in PDMS (Poly-dimethylsiloxane) by using soft lithography. A layer of SU-8 was patterned onto a 3″ Silicon wafer that acts as a master mold. The SU-8 photo-patterning process involves standard cleaning, spin coating, soft baking, exposure, development, and hard baking. After the master mold was fabricated, PDMS (10:1 prepolymer/curing agent) was poured onto the master mold and baked at 80^{oC} over 2 h for curing. The PDMS channel was then peeled off from the mold. A 5-mm hole and a 3-mm hole were then punched to form the inlet and outlet, respectively. The PDMS substrate was then aligned and bonded to the electrode chip after both substrates have undergone oxygen plasma treatment. The bonded chip was then baked at 70 °C for 40 min to form the irreversible bond. Our microfluidic channel had a width of 20 µm and height of 15 µm.

Optimization of Electrical parameters of the Impedance Spectroscope

Multiple configurations of the electrical parameters of the impedance spectroscope were tested for optimizing the detection of the DNA nanoballs. We performed experiments by passing Dynabeads™ MyOne™ in 1 x Phosphate Buffered Saline solution (PBS) for 10 minutes and then recording the response. The data was analyzed for baseline voltage, the signal-to-noise ratio, the number of beads detected, and the peak amplitude. All the measurements were done in the Faraday cage to reduce noise and interference from external sources. The electrical parameters were changed in a progressive fashion. We first increased the excitation voltage. The increase in excitation voltage results in a better signal. However, the electrodes break down at very high voltage and therefore we progressively increased the voltage until it reached the maximum allowable limit. We also changed the transimpedance gain, the bandwidth, and the excitation frequency to find the optimal electrical parameters for the detection of 1 μm particles in the microfluidic chip. Although we optimized the parameters for the detection of 1 μm beads, experiments showed that there was a similar improvement for detection of the DNA nanoballs.

Supplementary tables and figures

Name of primer	Sequence (5'-3')
As1 F3 FAM	/5-6-FAM/CGGTGGACAAATTGTCAC
As1 LF	TTACAAGCTTAAAGAATGTCTGAACACT
As1 LB	TTGAATTTAGGTGAAACATTTGTCACG
As1 B3	CTTCTCTGGATTTAACACACTT
As1e FIP	TCAGCACACAAAGCCAAAAATTTATTTTTCTGTGCAAAGGAAATTAAGGAG
As1e BIP	TATTGGTGGAGCTAAACTTAAAGCCTTTTCTGTACAATCCCTTTGAGTG
As1_LF_Two_repeat_ Compaction	TTACAAGCTTAAAGAATGTCTGAACACTAAATTACAAGCTTAAAGAATGTC TGAACACT
As1_LB Two_repeat_ Compaction	TTGAATTTAGGTGAAACATTTGTCACGAAATTGAATTTAGGTGAAACATTT GTCACG
As1_LF Two_repeat_ Compaction__InvdT	TTACAAGCTTAAAGAATGTCTGAACACTAAATTACAAGCTTAAAGAATGTC TGAACACT /3InvdT/
As1_LB Two_repeat_ Compaction__InvdT	TTGAATTTAGGTGAAACATTTGTCACGAAATTGAATTTAGGTGAAACATTT GTCACG /3InvdT/
As1_LF Three_repeat_ Compaction_InvdT	TTACAAGCTTAAAGAATGTCTGAACACTAAATTACAAGCTTAAAGAATGTC TGAACACTAAATTACAAGCTTAAAGAATGTCTGAACACT/3InvdT/
As1_LB Three_repeat_ Compaction_InvdT	TTGAATTTAGGTGAAACATTTGTCACGAAATTGAATTTAGGTGAAACATTT GTCACGAAATTGAATTTAGGTGAAACATTTGTCACG /3InvdT/

Table S1 :As1e SARS_CoV_2 RT LAMP and compaction primers

Configuration	Excitation Voltage (V)	Frequency (MHz)	Transimpedance Gain (kΩ)	Bandwidth (Hz)	Remarks
1	1	1	1	100	
2	1.5	1	1	100	
3	3	1	1	100	
4	5	1	1	100	
5	5	2	1	100	
6	5	5	1	100	Best Configuration
7	5	5	1	70	
8	5	6	1	40	
9	5	7	1	40	
10	0.3	5	10	70	
11	0.5	5	10	70	
12	10	5	1	40	Electrode Breakdown/ Hydrolysis

Table S2. Various configurations for electrical parameter optimizations. Configuration 6 yielded the best results. Results are shown in Figure S1.

Sample	N1 Ct (SARS-CoV-2)	S Ct (Omicron BA.1)	RNaseP Ct (internal control)	WGS lineage assignment
**1**	21.80	Undetermined	31.11	Omicron BA.2
**2**	19.22	21.83	29.61	Omicron BA.1
**3**	26.59	Undetermined	29.77	Omicron BA.2
**4**	23.65	26.78	31.78	Omicron BA.1
**5**	23.94	Undetermined	28.58	Omicron BA.2
**6**	21.44	Undetermined	29.69	Omicron BA.2
**7**	26.9	30.07	31.37	Omicron BA.1
**8**	22.73	Undetermined	30.22	Omicron BA.2
**9**	25.94	Undetermined	29.15	Omicron BA.2
**10**	27.82	Undetermined	34.55	Omicron BA.2
**11**	26.08	Undetermined	32.94	Omicron BA.2
**12**	21.37	Undetermined	30.20	Omicron BA.2
**13**	25.58	28.77	33.24	Omicron BA.1
**14**	25.24	Undetermined	32.58	Omicron BA.2
**15**	25.67	Undetermined	29.97	Omicron BA.2
**16**	17.21	Undetermined	28.06	Omicron BA.2
**17**	22.87	25.58	29.59	Omicron BA.1
**18**	21.56	Undetermined	29.34	Omicron BA.2
**19**	20.97	Undetermined	33.11	Omicron BA.2
**20**	21.41	Undetermined	27.10	Omicron BA.2
**21**	25.07	Undetermined	33.70	Omicron BA.2
**22**	19.16	22.32	30.58	Omicron BA.1
**23**	23.42	Undetermined	30.07	Omicron BA.2
**24**	24.37	Undetermined	31.70	Omicron BA.2
**25**	20.16	Undetermined	35.63	Omicron BA.2
**26**	26.50	Undetermined	30.01	Omicron BA.2

**27**	26.57	Undetermined	33.75	Omicron BA.2
**28**	22.80	26.45	27.20	Omicron BA.1
**29**	27.52	30.77	29.19	Omicron BA.1
**30**	24.51	Undetermined	29.25	Omicron BA.2

Table S3. Clinical patient sample data

**Name of primer**	**Sequence (5'-3')**
Myco_F3	CCTATCCGTATGGTGGATAACG
Myco_B3	GTCGGAAGCTCCTATGACAAT
Myco_FIP	ACCGGATCGATGTGTACTGAGA/ TTTT/ CCAACAAGAAGGCGTACTC
Myco_BIP	ATCCAACCGTCGGTCGGA/TTTT/ TTGATCGTCTCGGCTAGT
Myco_LF	GATCGAGCAAGCCATCTG
Myco_LB	TGATCAGCTCGGTCTTGTA
Myco_three_repeat compaction_LF	GATCGAGCAAGCCATCTGAAAGATCGAGCAAGCCATCTGAAAGATCGAGCAAGCCATCTG
Myco_three_repeat compaction LB	TGATCAGCTCGGTCTTGTAAAATGATCAGCTCGGTCTTGTAAAATGATCAGCTCGGTCTTG TA
HIV_LF	CTTTCCAGAGAAGCTTTGCT
HIV_LB	AGCAAAGATCATTAGGGATTAT
HIV_FIP	CTTGTATTACTACTGCCCCTTCACGATCCACTTTGGAAAGGACC
HIV_BIP	TGACATAAAAGTAGTGCCAAGAAGATTTTACAATCATCACCTGCCATC
HIV_F3	GGTTTATTACAGGGACAGCA
HIV_B3	ATCCTGTCTACTTGCCAC
HIV_three_repeat Compaction LF	CTTTCCAGAGAAGCTTTGCTAAACTTTCCAGAGAAGCTTTGCTAAACTTTCCAGAGAAGCT TTGCTA
HIV_three_repeat Compaction_LB	AGCAAAGATCATTAGGGATTATAAAAGCAAAGATCATTAGGGATTATAAAAGCAAAGATC ATTAGGGATTAT
Influenza_LF	ACTTGTCTTGGGGAATATCTC
Influenza_BIP	ATGCTGGAGCAAAAAGCT
Influenza_FIP	CGAGTCATGATTGGGCCATGACAGTGTCATCATTTGAAAGGTTT
Influenza_BIP	AAGGTGTAACGGCAGCATGTCCGAATTTCCTTTTTTAACTAGCCAT
Influenza_F3	GCTAAGAGAGCAATTGAGC
Influenza_B3	ATGTAGGATTTGCTGAGCT
Influenza_three_ repeat compaction__LF	ACTTGTCTTGGGGAATATCTCAAAACTTGTCTTGGGGAATATCTC AAAACTTGTCTTGGGGAATATCTC
Influenza_three_ repeat compaction_LF	ATGCTGGAGCAAAAAGCTAAAATGCTGGAGCAAAAAGCTAAAATGCTGGAGCAAAAAGC T
Beta_Lac_F3	TTGGAATGCTCACTTTACTGAA

Beta_Lac_B3	CGATATCGCGCGTCTGTC
Beta_Lac_FIP	TGCTTGGTTCGCCCGTTTAAGATGGCGTAGTTGTGCTCTGG
Beta_Lac_BIP	TTCCCAATAGCTTGATCGCCCTCCCACTTAAAGACTTGGTGT
Beta_Lac_LF	ATTGGTAAATCCTTGCTGCT
Beta_Lac_LB	TGGGCGTGGTTAAGGATGA
Beta_Lac_three_ repeat compaction_LF	ATTGGTAAATCCTTGCTGCTAAAATTGGTAAATCCTTGCTGCTAAAATTGGTAAATCCTTGC TGCT
Beta_Lac_three_ repeat compaction_LB	TGGGCGTGGTTAAGGATGAAAATGGGCGTGGTTAAGGATGAAAATGGGCGTGGTTAAGGA TGA

Table S4. Other targets RT LAMP and compaction primers

Primer name	Sequence (5’-3’)
Ultramer_1__myco	ACCTCCATGGTCCTCGACGCGATCGAGCAAGCCATCTGGACCCGCCAACAAGAAGGCGTA CTCGACCTGAAAGACGTTATCCACCATACGGATAGGGGATCTCAGTACACATCGATCCGGT TCAGCGAGCGGCTCGCCGAGGCAGGCATC
Ultramer_2__myco	CCGCCAGGGCTTGCCGGGTTTGATCAGCTCGGTCTTGTATAGGCCGTTGATCGTCTCGGCT AGTGCATTGTCATAGGAGCTTCCGACCGCTCCGACCGACGGTTGGATGCCTGCCTCGGCGA GCCGCTCGCTGAACCGGATCGATGTGTA
For_T7_Myco	ATCGATTAATACGACTCACTATAGGGACCTCCATGGTCCTCGACGC
Rev_T3_Myco	TACGTTAATTAACCCTCACTAAAGCCGCCAGGGCTTGCC
Ultramer_1_HIV	ACAAAAATTCAAAATTTTCGGGTTTATTACAGGGACAGCAGAAATCCACTTTGGAAAGGA CCAGCAAAGCTCCTCTGGAAAGGTGAAGGGGCAGTAGTAATACAAGATAATAGTGACATA
Ultramer_2_HIV	CTCATCCTGTCTACTTGCCACACAATCATCACCTGCCATCTGTTTTCCATAATCCCTAATGA TCTTTGCTTTTCTTCTTGGCACTACTTTTATGTCACTATTATCTTGTATTACTACTGC
For_T7_HIV	ATCGATTAATACGACTCACTATAGGGACAAAAATTCAAAATTTTCGGGTTTATTA
Rev_T3_HIV	TACGTTAATTAACCCTCACTAAAGCTCATCCTGTCTACTTGCCA
Ultramer_1_ Influenza	GATTTCATCGATTATGAGGAGCTAAGAGAGCAATTGAGCTCAGTGTCATCATTTGAAAGGT TTGAGATATTCCCCAAGACAAGTTCATGGCCCAATCATGACTCGAACAAAGGTGTAACGG CAGCATGTCCTCATGCTGG
Ultramer_2_ Influenza	GACTTCTTTCCCTTTATCATTAATGTAGGATTTGCTGAGCTTTGGGTATGAATTTCCTTTTTT AACTAGCCATATTAAATTTTTGTAGAAGCTTTTTGCTCCAGCATGAGGACATGCTGCCGTT ACACCTTTGTTCGAGTCA
For_T7_Influenza	ATCGATTAATACGACTCACTATAGGGGATTTCATCGATTATGAGGA
Rev_T3_Influenza	TACGTTAATTAACCCTCACTAAAGGACTTCTTTCCCTTTATCAT
Ultramer_1_ Beta_Lac	ACCTCCATGGTCCTCGACGCTTGGAATGCTCACTTTACTGAACATAAATCACAGGGCGTAG TTGTGCTCTGGAATGAGAATAAGCAGCAAGGATTTACCAATAATCTTAAACGGGCGAACC AAGCATTTTTACCCGCATCTACCTTTAAAA
Ultramer_2_ Beta_Lac	ATGATCGCGATTCCAAGTGGCGATATCGCGCGTCTGTCCATCCCACTTAAAGACTTGGTGT TCATCCTTAACCACGCCCAAATCGAGGGCGATCAAGCTATTGGGAATTTTAAAGGTAGATG CGGGTAAAAATGCTTGGTTCGCCCGTTT
For_T7_Beta_Lac	ATCGATTAATACGACTCACTATAGGGACCTCCATGGTCCTCGACGC
Rev_T3_Beta_Lac	TACGTTAATTAACCCTCACTAAAGATGATCGCGATTCCAAGTGGCGATA

Table S5. Ultramer sequences and related PCR primers

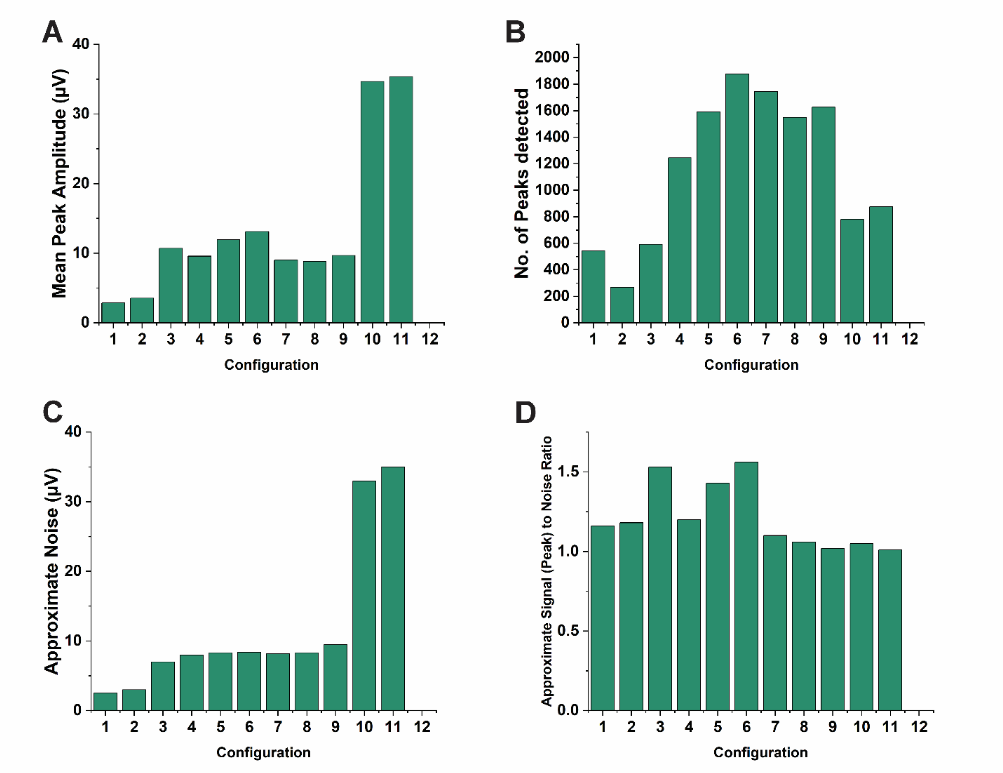

Fig. S1: Comparison of the various LIA configurations – configuration parameters shown in Table S2 (A) Mean Peak Amplitude **(µV) (B)** Number of peaks detected **(C)** Approximate Noise (µV) **(D)** Approximate Signal-to-Noise Ratio (SNR)

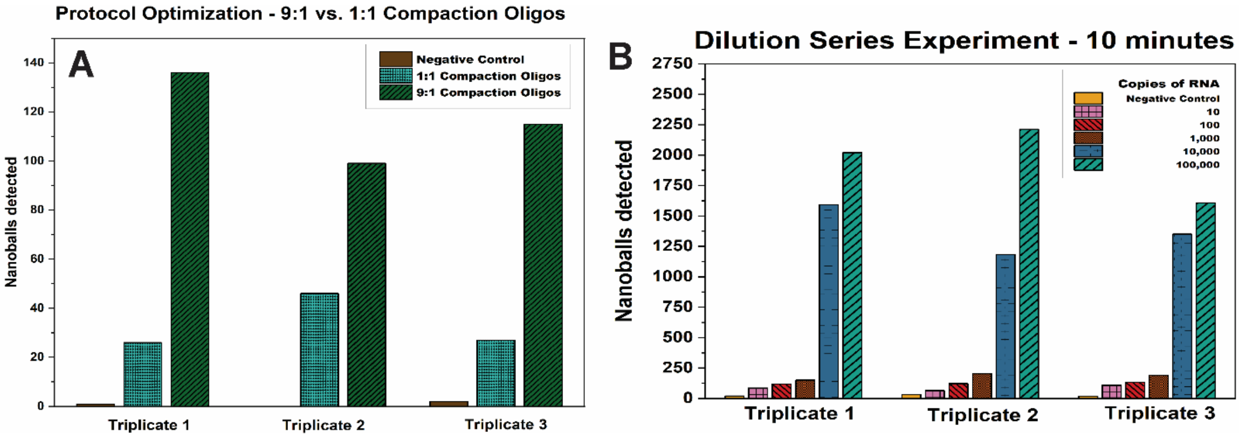

Fig. S2: Comparison of the DNA nanoballs detected before and after optimization of Electrical parameters. (A) DNA Nanoballs detected before optimization of electrical parameters. **(B)** DNA Nanoballs detected after optimization of electrical parameters.

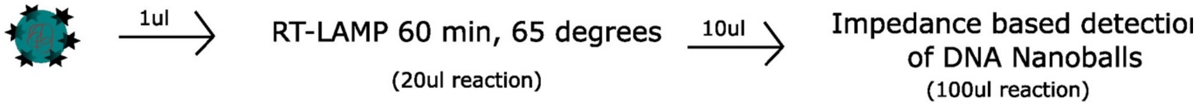

Fig. S3: Simplified outline of full experimental protocol. A 60min RT-LAMP reaction using target specific LAMP primers and compaction oligos produces DNA Nanoballs which are subsequently detected.
